# Metabolic modulation predicts heart failure tests performance

**DOI:** 10.1371/journal.pone.0218153

**Published:** 2019-06-20

**Authors:** Daniel Contaifer, Leo F. Buckley, George Wohlford, Naren G. Kumar, Joshua M. Morriss, Asanga D. Ranasinghe, Salvatore Carbone, Justin M. Canada, Cory Trankle, Antonio Abbate, Benjamin W. Van Tassell, Dayanjan S. Wijesinghe

**Affiliations:** 1 Department of Pharmacotherapy and Outcomes Sciences, School of Pharmacy, Virginia Commonwealth University, Richmond, Virginia, United States of America; 2 Department of Microbiology, School of Medicine, Virginia Commonwealth University, Richmond, Virginia, United States of America; 3 Department of Physical and Biological Sciences, Amarillo College, Amarillo, Texas, United States of America; 4 Pauley Heart Center, Virginia Commonwealth University, Richmond, Virginia, United States of America; 5 Department of Internal Medicine, School of Medicine, Virginia Commonwealth University, Richmond, Virginia, United States of America; 6 Da Vinci Center, Virginia Commonwealth University, Richmond, Virginia, United States of America; 7 Institute for Structural Biology Drug Discovery and Development (ISB3D), School of Pharmacy, Virginia Commonwealth University, Richmond, Virginia, United States of America; Ospedale del Cuore G Pasquinucci Fondazione Toscana Gabriele Monasterio di Massa, ITALY

## Abstract

The metabolic changes that accompany changes in Cardiopulmonary testing (CPET) and heart failure biomarkers (HFbio) are not well known. We undertook metabolomic and lipidomic phenotyping of a cohort of heart failure (HF) patients and utilized Multiple Regression Analysis (MRA) to identify associations to CPET and HFBio test performance (peak oxygen consumption (Peak VO_2_), oxygen uptake efficiency slope (OUES), exercise duration, and minute ventilation-carbon dioxide production slope (VE/VCO_2_ slope), as well as the established HF biomarkers of inflammation C-reactive protein (CRP), beta-galactoside-binding protein (galectin-3), and N-terminal prohormone of brain natriuretic peptide (NT-proBNP)). A cohort of 49 patients with a left ventricular ejection fraction < 50%, predominantly males African American, presenting a high frequency of diabetes, hyperlipidemia, and hypertension were used in the study. MRA revealed that metabolic models for VE/VCO_2_ and Peak VO_2_ were the most fitted models, and the highest predictors’ coefficients were from Acylcarnitine C18:2, palmitic acid, citric acid, asparagine, and 3-hydroxybutiric acid. Metabolic Pathway Analysis (MetPA) used predictors to identify the most relevant metabolic pathways associated to the study, aminoacyl-tRNA and amino acid biosynthesis, amino acid metabolism, nitrogen metabolism, pantothenate and CoA biosynthesis, sphingolipid and glycerolipid metabolism, fatty acid biosynthesis, glutathione metabolism, and pentose phosphate pathway (PPP). Metabolite Set Enrichment Analysis (MSEA) found associations of our findings with pre-existing biological knowledge from studies of human plasma metabolism as brain dysfunction and enzyme deficiencies associated with lactic acidosis. Our results indicate a profile of oxidative stress, lactic acidosis, and metabolic syndrome coupled with mitochondria dysfunction in patients with HF tests poor performance. The insights resulting from this study coincides with what has previously been discussed in existing literature thereby supporting the validity of our findings while at the same time characterizing the metabolic underpinning of CPET and HFBio.

## Introduction

The prevalence of heart failure (HF) has increased over time in the aging population. In people older than 20, the incidence of HF has increased from 5.7 million Americans between 2009 and 2012 to 6.5 million Americans in 2011. Despite aggressive measures to improve HF management, the five-year mortality rate of HF patients remains approximately 40%—comparable to many forms of cancer[1,2]. Investigations into diagnosis of HF has revealed promising cardiopulmonary tests and biomarkers that allow better disease management following diagnosis[3,4]. Combining patient’s metabolic profiles from HF compromised organs and tissues with HF tests has been demonstrated to provide physicians with an efficient source of clinical information used to both manage and diagnose patients[5]. However, the complex association of these HF tests with changes in the peripheral metabolism of compromised individuals is still under investigation and has failed to reveal the value of circulating metabolites as HF biomarkers[6].

Impaired cardiorespiratory fitness measured during cardiopulmonary exercise testing (CPET) is a hallmark manifestation of heart failure[7] and exercise training reduces all-cause mortality in patients with heart failure and reduced left ventricular ejection fraction (HFrEF)[8]. As such, cardiopulmonary exercise testing (CPET) is an evidence-supported assessment technique routinely used in the functional diagnosis of HF, prognostic evaluation of patients with chronic Heart Failure (CHF), and is also clinically relevant to supplement other clinical data in patients’ screening for heart transplantation[9,10]. Similarly, HF biomarkers (HFBio) such as NT-proBNP, Galectin-3, and C-reactive protein (CRP) have shown promising results as predictors of mortality[9,11]. However, a potential disadvantage of the CPET and HFBio use is that the evaluation of their response alone is insufficient to indicate risk of heart disease nor is it enough to diagnose a heart problem[12,13].

It is now known that cardiac and peripheral metabolic abnormalities may contribute to the pathogenesis of heart failure[14]. Studies of the metabolic profile of HF patients have indicated a rich metabolic modulation that can be identified and used as putative biomarkers[15–17]. However, little is known about the relationship of cardiorespiratory fitness and global metabolic profile in HFrEF. Efforts to correlate clinical and metabolic data are still necessary to fully integrate metabolomics as a translational medicine apparatus[18]. We hypothesized that deep metabolomic and lipidomic phenotyping would reveal novel metabolic and lipid mediators of cardiorespiratory fitness in patients with HFrEF. To test this hypothesis, we employed a Multiple Regression Analysis (MRA) study on the association of both CPET and HFBio with respects to the plasma lipidome and the metabolome of HF patients demonstrating that the metabolic modulation in HF patients depends on the tests’ performance. Although MRA does not imply causality for the HF performance, this analysis intends to reveal the metabolic changes underlying the complex HF pathology to maximize the relevance of the HF tests and its potential for HF outcome prognosis.

## Materials and methods

### Patients

This research was approved by the Virginia Commonwealth University (VCU) Institutional Review Board (VCU IRB number HM15339). Written informed consent was obtained from all participants. A post-hoc analysis was performed on patients who were enrolled in the REDHART study[19], which included patients with a left ventricular ejection fraction < 50% and high-sensitivity C-reactive protein (hsCRP) ≥2 mg/L and who were recently discharged after a hospitalization for HF. The present analysis includes plasma samples collected from 49 patients at baseline prior to randomization, since we were interested in the baseline analysis only without the cofounding randomization for drugs and place groups of the original study. Because the study was a post-hoc analysis, there were only enough plasma samples from 49 patients out of 52 in the REDHART study. Also, there were not health matched control group in the REDHART study, the reason for the absence of comparison with health subject in our analysis. The design and results of REDHART have been reported previously[19]. All patients underwent maximal aerobic exercise testing using a metabolic cart and a treadmill according to a conservative ramping treadmill protocol. Patients exercised to exhaustion (peak respiratory exchange ratio ≥1.00 and preferably ≥1.10) and those who were unable to complete the cardiopulmonary exercise test were excluded. The core CPET lab at University of Illinois at Chicago conducted all analyses of cardiopulmonary exercise test variables (peak oxygen consumption, oxygen uptake efficiency slope, exercise duration, minute ventilation-carbon dioxide production [VE/VCO_2_] as described previously[19].

### Lipidomic and metabolomic data acquisition

#### Lipid and metabolite extraction for LC-MS/MS analyses

Blood plasma lipids extraction was carried out using a biphasic solvent system of cold methanol, methyl *tert*-butyl ether (MTBE), and water with some modifications[20]. In detail, 225 μL of cold methanol containing a mixture of odd chain and deuterated lipid internal standards [lysoPE(17:1), lysoPC(17:0), PC(12:0/13:0), PE(17:0/17:0), PG(17:0/17:0), sphingosine (d17:1), d7 cholesterol, SM(17:0), C17 ceramide, d3 palmitic acid, MG(17:0/0:0/0:0), DG(18:1/2:0/0:0), DG(12:0/12:0/0:0), and d5 TG(17:0/17:1/17:0)] was added to a 20 μL blood plasma aliquot in a 1.5 mL polypropylene tube, and then vortexed. Next, 750 μL of cold MTBE was added, followed by vortexing and shaking with an orbital mixer. Phase separation was induced by adding 188 μL of MS-grade water. Upon vortexing (20s) the sample was centrifuged at 12,300 rpm for 2 min. The upper organic phase was collected in two 300 μL aliquots and evaporated with a vapor trap. Dried extracts were resuspended using 110 μL of a methanol/toluene (9:1, v/v) mixture containing CUDA (50 ng/ml; internal standard for quality control of injection) with support of vortexing (10 s), and centrifuged at 800 rpm for 5 min, followed by transferring 100 uL of the supernatant into autosampler vial with an insert. An aliquot of 125 μL of the lower polar layer was evaporated to dryness in a SpeedVac, resuspended in acetonitrile, and used for metabolite analysis via HILIC LC-MS/MS method.

#### Metabolomics: GC-MS metabolite extraction

30μl of plasma sample was added to a 1.0 mL of pre-chilled (-20°C) extraction solution composed of acetonitrile, isopropanol and water (3: 3: 2, v/v/v). Sample were vortexed and shaken for 5 min at 4°C using the Orbital Mixing Chilling/Heating Plate. Next, the mixture was centrifuged for 2min at 14,000 rcf. 450μL of the supernatant was dried with cold trap concentrator. The dried aliquot was then reconstituted with 450 μL acetonitrile:water (50:50) solution and centrifuged for 2 min at 14,000 rcf. The supernatant was transferred to a polypropylene tube and subjected to drying in a cold trap. The process of derivatization began with addition of 10 μL of 40 mg/mL Methoxyamine hydrochloride solution to each dried sample and standard. Samples were shaken at maximum speed at 30 °C for 1.5 hours. Then, 91 μL of MSTFA + FAME mixture was added to each sample and standard and capped immediately. After shaking at maximum speed at 37 °C, the content was transferred to glass vials with inserts and cap immediately.

#### Lipids: LC-MS/MS conditions

Untargeted lipid analysis was obtained with Sciex TripleTOF 6600 coupled to Agilent 1290 LC. Lipids were separated on an Acquity UPLC CSH C18 column (100 × 2.1 mm; 1.7 μm) (Waters, Milford, MA, USA). The column was maintained at 65 °C and the flow-rate of 0.6 mL/min. The mobile phases consisted of (A) 60:40 (v/v) acetonitrile:water with 10 mM ammonium acetate and (B) 90:10 (v/v) isopropanol:acetonitrile with 10 mM ammonium acetate. The separation was conducted following a stepwise gradient: 0–2 min 15–30% (B), 2–2.5 min 48% (B), 2.5–11 min 82% (B), 11–11.5 min 99% (B), 11.5–12 min 99% (B), 12–12.1 15% (B), 12–14 min 15% (B). Negative and positive electrospray ionization (ESI) modes were applied with nitrogen serving as the desolvation gas and the collision gas. The parameters for detection of lipids were as follows: Curtain Gas: 35; CAD: High; Ion Spray Voltage: 4500 V; Source Temperature: 350°C; Gas 1: 60; Gas 2: 60; Declustering Potential: +/- 80V, and collision energies +/- 10.

#### Metabolites HILIC: LC-MS/MS conditions

Detection of water soluble plasma metabolites was carried out on Sciex TripleTOF 6600 coupled to Agilent 1290 LC. Analytes were separated on an Acquity UPLC BEH Amide Column, 130Å, 1.7 μm, 2.1 mm X 150 mm (Waters, Milford, MA, USA). The column was maintained at 45 °C and the flow-rate of 0.4 mL/min. The mobile phases consisted of (A) water with 10 mM ammonium formate, 0.125% formic acid, and (B) acetonitrile:water 90:10 (v/v) with 10 mM ammonium formate, 0.125% formic acid. The analytes separation was conducted following a stepwise gradient: 0–2 min 100% (B), 2–7.7 min 70% (B), 7.7–9.5 min 40% (B), 9.5–10.25 min 30% (B), 10.25–12.75 min 100% (B), 12.75–16.75 100% (B). A sample volume of 1 μL and 3 μL were injected for positive and negative mode, respectively. Negative and positive electrospray ionization (ESI) modes were applied with nitrogen serving as the desolvation gas and the collision gas. The parameters for detection of lipids were as follows: Curtain Gas: 35; CAD: High; Ion Spray Voltage: 4500 V; Source Temperature: 300°C; Gas 1: 60; Gas 2: 60; Declustering Potential: +/- 80V, and collision energies +/- 10.

#### Metabolites: GC-MS conditions

A Leco Pegasus IV time of flight mass spectrometer coupled with Agilent 6890 GC equipped with a Gerstel automatic liner exchange system (ALEX) that included a multipurpose sample (MPS2) dual rail, and a Gerstel CIS cold injection system (Gerstel, Muehlheim, Germany) was used to complement HILIC metabolite analysis. The transfer line was maintained at 280 °C. Chromatography separation was achieved on a 30 m long, 0.25 mm i.d. Rtx-5Sil MS column (0.25 μm 95% dimethyl 5% diphenyl polysiloxane film) with the addition of a 10 m integrated guard column (Restek, Bellefonte PA) with helium (99.999%; Airgas, Radnor, PA, U.S.A.) at a constant flow of 1 mL/min. The oven temperature was held constant at 50°C for 1 min and then ramped at 20°C/min to 330°C at which it is held constant for 5 min. The GC temperature program was set as follows: 50°C to 275°C final temperature at a rate of 12 °C/s and hold for 3 minutes. The injection volume was 1 μL in splitless mode at 250 °C. Electron impact ionization at 70V was employed with an ion source temperature of 250°C. The scan mass ranged from 85 to 500 Da with acquisition rate of 17 spectra/second.

### Statistical analysis

**Fig 1:**
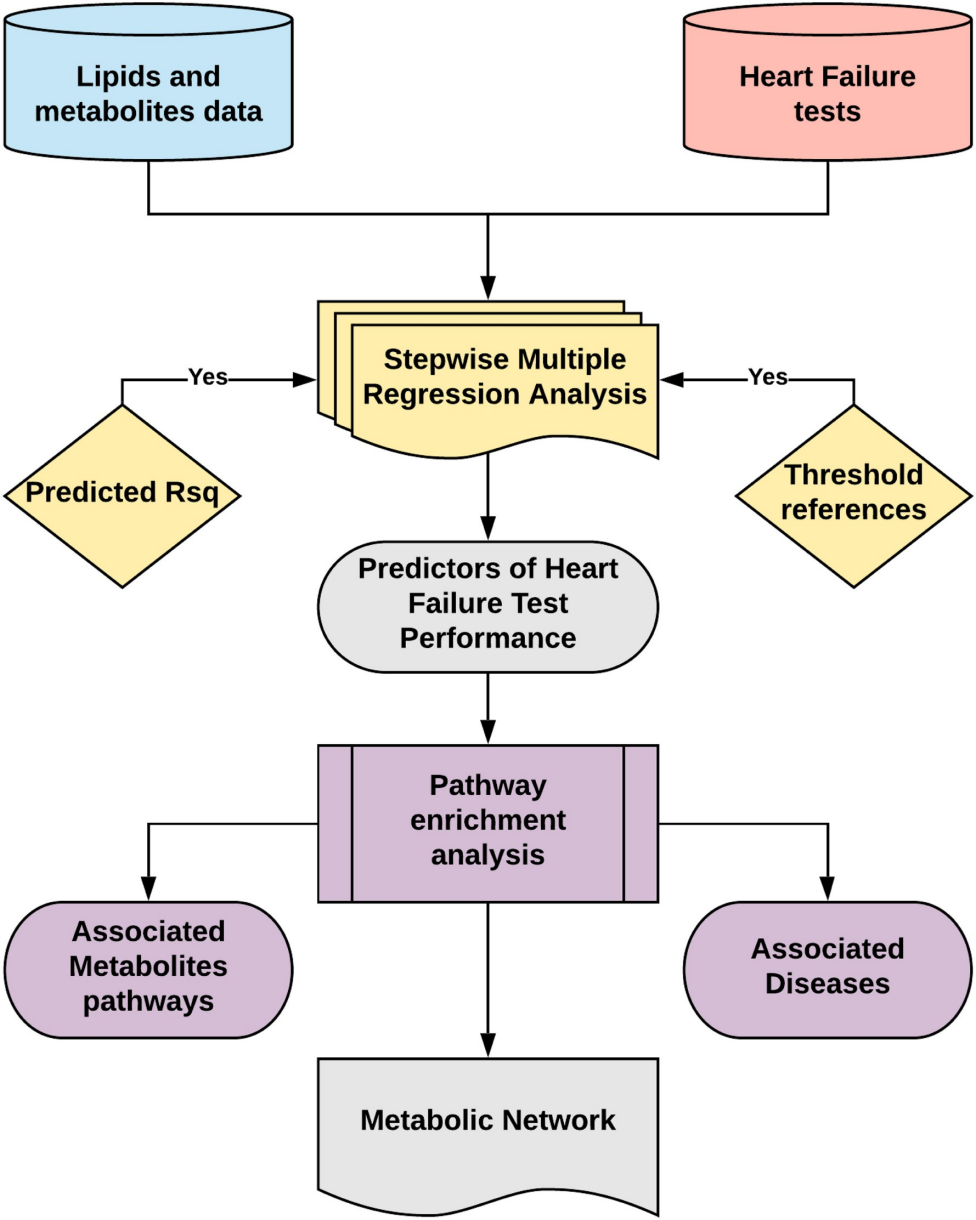
Statistical analysis workflow outlining the steps taken to find predictors of HF test performance using metabolomic and lipidomic data. Stepwise MRA was validated with bootstrap 95% confidence interval of the main regression estimates. Search of published threshold references confirmed the clinical importance of the models. Pathway enrichment analysis revealed the main metabolic pathways and associated diseases enriched using the set of metabolites predicting HF performance. A metabolic network was derived from the analysis confirming several metabolic dysfunctions related to HF described in the literature.

The statistical workflow used to find predictors of the CPET and HFBio tests, and reveal the metabolic pathways associated with HF, is depicted in Fig 1. Lipidomic and metabolomic data were presented as peak heights normalized by mTIC, a form of sample normalization[21]. Using mTIC allows for the merger of the two databases. Outliers were eliminated using the Interquartile range (IQR) from a box-and-whisker plot with Turkey’s method detecting outliers as any value for the determined test outside of 1.5 times the IQR[22]. Metabolic data were filtered to exclude detected prescribed drugs, and from the lipidomic data only phospholipids, with detected fatty acids composition, were used in the analysis. The final 316 lipids and 167 metabolites were used in the analysis.

We utilized stepwise multiple regression analysis and Standard Least Squares methods to identify the set of metabolites which best associated with measures of cardiorespiratory fitness peak oxygen consumption (Peak VO2), oxygen uptake efficiency slope (OUES), exercise duration, and minute ventilation-carbon dioxide production slope (VE/VCO_2_ slope), as well as the established HF biomarkers of inflammation C-reactive protein (CRP), beta-galactoside-binding protein (galectin-3), and N-terminal prohormone of brain natriuretic peptide (NT-proBNP). Covariate and cofounders bias was not taken in account in the MRA because the aim of the study was not focused in causal analysis, but rather in the lipid and metabolite associations with HF tests to reveal the underlying metabolic modulation. Therefore, unadjusted regression models were utilized in this study, and no control group was included.

MRA method was applied using the forward selection to enter the terms with the lowest p-values, allowing that at each step the candidate variable that increased the adjusted R^2^ the most was selected, until the model reached an adjusted R^2^ ≥ 0.8 and all the predictors had a statistically significant effect (p<0.05). All models reached a statistical significance of p<0.001. *To avoid bias* due to presence of outliers, subjects presenting extreme values in the HF tests were excluded for the specific model. The number of observations and predictors in each model is depicted in Table 1. To enable comparison, the different models were standardized by mean centering of their coefficients, and the mean response of their dependent variables was estimated. The Root Mean Square Error (RMSE) was obtained and used as an estimate of each model’s fit. Coefficient of Variation (CV) was calculated as the ratio of RMSE to mean response of the dependent variable with its result suggesting good model fit and allowing for intermodal comparison. We also performed a statistical cross-validation to determine the predicted R^2^. This procedure is executed by removing a data point from the dataset, calculating the regression equation, and then evaluating how well the model predicts the missing observation. This is repeated for all data points in the dataset and a predicted R^2^ is generated.

**Table 1:** Estimates of cardiorespiratory fitness and HF biomarkers and comparison with literature threshold references.

Test	Mean response	R^2^ adjusted	RMSE	CV	Threshold References
**OUES** **10 predictors** **(n = 47)**	1.68 (1.53–1.83)	0.857 (0.742–0.896)	0.195 (0.186–0.235)	0.12	1.47 Davies *et al*[17]
**Exercise duration** **11 predictors** **(n = 49)**	6.99 minutes (6.24–7.74)	0.864 (0.780–0.892)	0.973 (0.933–1.117)	0.14	7.50 minutes Florea *et al*[18]
**Peak VO_2_** **15 predictors** **(n = 48)**	13.81 mL·kg^-1^·min^-1^ (12.94–14.71)	0.864 (0.775–0.886)	1.145 (1.120–1.353)	0.08	14.00 mL·kg^-1^·min^-1^ Arena *et al*[19]
**VE/VCO_2_ Slope** **13 predictors** **(n = 45)**	33.83 (32.08–35.61)	0.863 (0.756–0.896)	2.270 (2.183–2.705)	0.07	32.90 Shen *et al*[20]
**NT-proBNP** **12 predictors** **(n = 46)**	1550 pg/mL (1244–1958)	0.870 (0.710–0.906)	442 (430–502)	0.29	≥900 pg/mL (50–75 years), ≥1800 pg/mL pg/mL (>75 years) Shah *et al*[21]
**Galectin-3** **12 predictors** **(n = 46)**	19.90 ng/mL (18.00–22.11)	0.868 (0.766–0.903)	2.576 (2.365–3.178)	0.13	19.00 ng/mL Carrasco-Sanchez *et al*[22]
**CRP** **15 predictors** **(n = 45)**	6.45 mg/L (5.11–8.21)	0.857 (0.725–0.890)	2.006 (1.936–2.323)	0.31	6.91 mg/L Mommersteeg *et al*

The mean response are values of the CPET and HFBio calculated from the regression parameters and a given value of the predictors that best fit the model. RMSE is presented as an absolute measure of fit in the same unit as the mean response. CV was calculated using the rate of RMSE by the mean response. Threshold references for test prediction of CHF outcome is presented as a measure of comparison of the mean response with peer reviewed publications. Bootstrap 95% CI are presented as a measure of validation, and are generally larger than 95% CI calculated from the actual data. Peak VO_2_ = peak oxygen consumption; OUES = oxygen uptake efficiency slope; VE/VCO_2_ = minute ventilation-carbon dioxide production; NT-proBNP = N-terminal pro-B-type natriuretic peptide; CRP = C-reactive protein; L/min = liters per minute; RMSE = root mean square error; CV = coefficient of variation.

To discover the metabolic pathways associated with HF, two methods of pathway enrichment analysis were performed. The first method was a Metabolic Pathway Analysis based on an over representation analysis with Fisher’s exact test algorithm to detect which metabolites were over-represented and significantly enriched. Coupled with this method, a pathway topological analysis with an out-degree centrality algorithm was used to measure the centrality of a metabolite in a metabolic pathway, estimating the mean importance of each matched metabolite impacting the pathway. The second method was a Metabolite Set Enrichment Analysis[23] that allows the incorporation into the analysis of pre-existing biological knowledge contained in metabolite-set libraries from studies of human metabolism. The analysis facilitated hypothesis generation and aided in interpretation of the metabolic models. Metabolite Set Enrichment Analysis used a reference metabolome from metabolite-set libraries to calculate a background distribution, and determine if the matched metabolite set in the model is more enriched for certain metabolites compared to random chance. The selection of pathway metabolites was based on the Kyoto Encyclopedia of Genes and Genomes (KEGG). To perform the study’s statistical analysis, multivariate linear regression was analyzed with JMP14Pro, and pathway enrichment analysis was performed with MetaboAnalyst 4.0.

### Results

**Fig 2:**
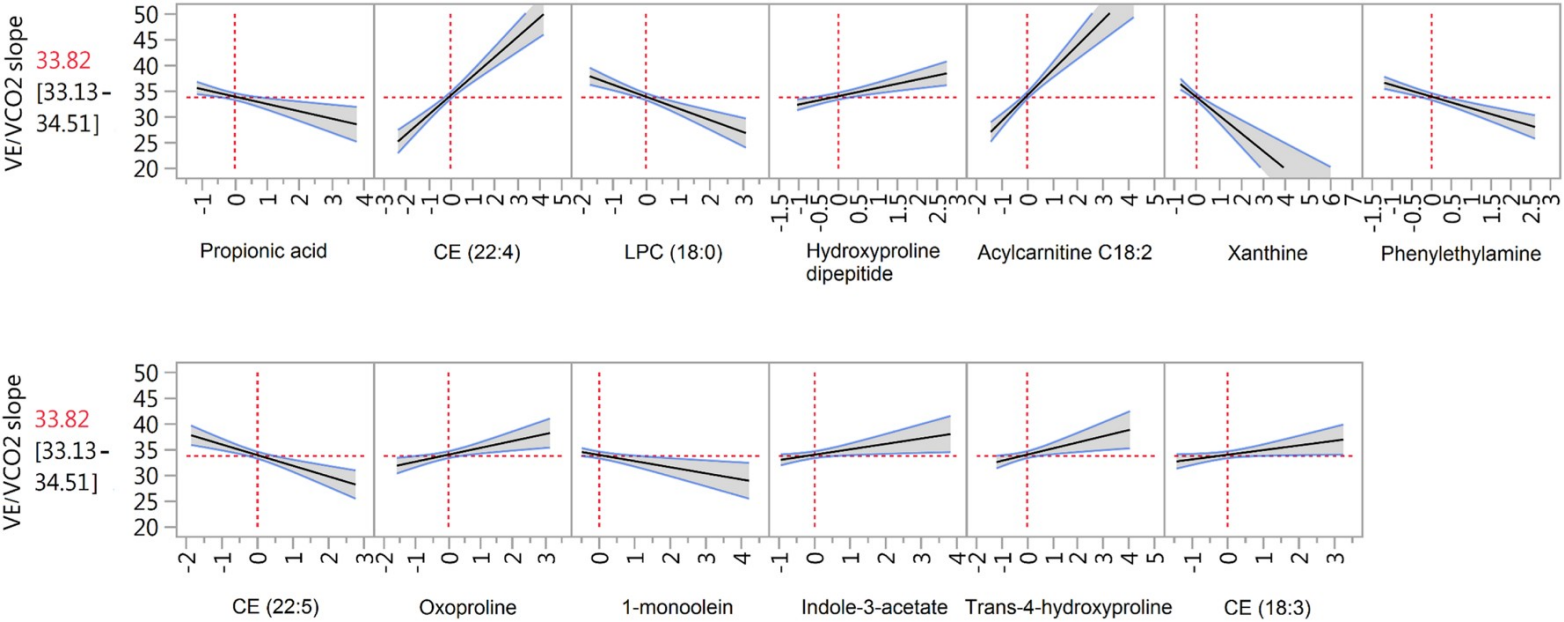
Prediction plot of VE/VCO_2_ shows the changes in expected VE/VCO_2_ slope value when the predictor’s levels change. When there are higher values of CE (22:4), CE (18:3) Acylcarnitine C18:2, hydroxyproline dipeptide, oxoproline,trans-4-hydroxyproline, and indole-3-acetate, as well as lower values of CE (22:5), LPC (18:0), 1-monoolein, propionic acid, xanthine, and phenylethylamine, the CPET test predicts poor performance. CE = cholesterol ester, LPC = lysophosphatidylcholine.

The study cohort included mostly African Americans with diabetes, hyperlipidemia, and hypertension (Table 2), characterizing this particular HF population for this single center study. The metabolic modulation underlying the HF test performance of this particular cohort is the main finding in our study. This modulation was revealed based in the evaluation of how the variation of the predictor’s values affect the HF tests performance in the MRA model. Using as example the prediction plot of VE/VCO2 (Fig 2), when there are higher values of CE (22:4), CE (18:3), Acylcarnitine C18:2, hydroxyproline dipeptide, oxoproline, trans-4-hydroxyproline, and indole-3-acetate, as well as lower values of CE (22:5), LPC (18:0), 1-monoolein, propionic acid, xanthine, and phenylethylamine, VE/VCO2 slope is above the mean response, which indicates poor performance. The higher the value of the coefficient, the higher the slope of the plot line, indicating the sensitivity of changes of the predictor value to estimate the test performance. The predictors of all HF tests performance are listed in Table 3 by order of the highest to the lowest coefficient absolute values. The highest predictors’ coefficients found with MRA were Acylcarnitine C18:2, palmitic acid, citric acid, asparagine, and 3-hydroxybutiric acid. All of our models rendered a predicted R^2^ higher than 0.7 in the cross-validation (S1 Fig). The 73 predictors taken together give an overall view of the metabolic state of HF patients, since the tests encompass cardiovascular and respiratory physiology.

**Table 2:** Demographics of the study cohort.

Covariates	Estimates
Age (median and IQR)	57 [52.5–64.5]
Male (%)	37 (75.5)
African American (%)	39 (79.6)
Coronary artery disease (%)	18 (36.7)
Diabetes (%)	28 (57.1)
Hyperlipidemia (%)	31 (63.3)
Hypertension (%)	46 (93.9)
Left ventricular ejection fraction (%)	32.1 ± 9.5
Left ventricular end-diastolic volume (mL)	184.0 ± 62.5
Left ventricular end-systolic volume (mL)	127.0 ± 52.3
Duke Activity Status Score	28.4 ± 16.6
Minnesota Living with Heart Failure Questionnaire	58.6 ± 19.3
Oxygen Uptake Efficiency Slope	1.8 ± 0.7
Peak VO_2_ (mL·kg^-^1·min^-1^)	14.0 ± 3.4
VE/VCO_2_ Slope	35.1 ± 7.3
Exercise Duration (minutes)	7.0 ± 2.6
NT-proBNP (pg·mL^-1^)	2650.3 ± 5475.1
C-reactive protein (mg·L^-1^)	8.2 ± 7.9
Galectin-3 (ng·mL^-1^)	21.4 ± 9.3

Estimate’s data are presented as percentage or mean ± standard deviation. IQR = Interquartile range; mL = milliliter; min = minutes; Peak VO_2_ = peak oxygen consumption; OUES = oxygen uptake efficiency slope; VE/VCO_2_ = minute ventilation-carbon dioxide production; NT-proBNP = N-terminal pro-B-type natriuretic peptide.

**Table 3:** Predictors of cardiorespiratory fitness and traditional HF biomarkers selected by multivariate linear regression.

CPET	Predictors	Stand. Coefficients	p-value
**Peak VO_2_**	Methionine	0.592	0.0001
	Quinic acid	-0.563	0.0001
	Glycine	-0.497	0.0001
	PI(18:0/20:4)	-0.388	0.0001
	Glycerol-3-galactoside	0.379	0.0001
	Lactic acid	-0.357	0.0001
	Serine	-0.292	0.0001
	Lysine	-0.273	0.0007
	Gluconic acid	-0.258	0.0014
	Glycerol-alpha-phosphate	0.257	0.0005
	CE (22:4)	-0.217	0.0021
	Glycolic acid	-0.21	0.0067
	GlcCer(NS)(d18:1/16:0)	-0.206	0.0055
	Myo-inositol	0.195	0.0071
	Adipic acid	-0.186	0.0125
**VE/VCO_2_**	Acylcarnitine C18:2	0.804	0.0001
	CE (22:4)	0.615	0.0001
	Xanthine	-0.57	0.0001
	LPC (18:0)	-0.373	0.0001
	Phenylethylamine	-0.367	0.0001
	CE (22:5)	-0.337	0.0001
	Hydroxyproline dipeptide	0.263	0.0003
	Indole-3-propionic acid	-0.233	0.0023
	Oxoproline	0.218	0.0037
	Trans-4-hydroxyproline	0.195	0.0087
	1-monoolein	-0.193	0.0053
	Indole-3-acetate	0.171	0.0237
	CE (18:3)	0.148	0.0425
**Exercise duration**	Citric acid	0.833	0.0001
	Lauric acid	-0.681	0.0001
	1-monostearin	-0.659	0.0001
	Malic acid	-0.618	0.0001
	Myristic acid	0.598	0.0001
	Glycerol	-0.561	0.0001
	Leucine	0.508	0.0001
	CE (18:1)	-0.325	0.0001
	LPC (18:0)	0.295	0.0001
	CE (22:4)	-0.257	0.0001
	Glycerol-alpha-phosphate	0.142	0.0192
**OUES**	Quinic acid	-0.422	0.0001
	Linolenic acid	0.418	0.0001
	Valine	0.401	0.0001
	PC(18:2/20:5)	0.344	0.0001
	DG(16:0/16:0)	0.327	0.0001
	PI(16:0/18:2)	-0.291	0.0001
	Hippuric acid	0.228	0.0009
	Glutamate	0.217	0.0047
	2-hydroxyhippuric acid	-0.197	0.0092
	Lauric acid	-0.188	0.0062
**CRP**	Palmitic acid	1.237	0.0001
	Heptadecanoic acid	-0.69	0.0001
	Cer(NS)(d18:1/16:0)	0.554	0.0001
	Pyrrole-2-carboxylic acid	0.519	0.0001
	CE (16:1)	-0.433	0.0001
	Indole-3-lactate	0.332	0.0001
	Ribose	0.291	0.0001
	Stearic acid	-0.288	0.0148
	Cholesterone	-0.237	0.0075
	1-monostearin	0.237	0.0008
	PI(16:0/20:4)	0.22	0.0095
	Isoleucine	0.215	0.0048
	Pelargonic acid	-0.199	0.0044
	1-monoolein	-0.166	0.0354
	3-aminoisobutyric acid	-0.148	0.0481
**Galectin-3**	Asparagine	-0.92	0.0001
	3-hydroxybutyric acid	-0.876	0.0001
	Cysteine	0.539	0.0001
	CE (20:4)	-0.438	0.0001
	Threonine	0.425	0.0004
	CE (18:1)	0.417	0.0001
	FA (24:1)	0.358	0.0001
	Tryptophan	-0.305	0.0002
	PC(18:2/20:5)	-0.279	0.0019
	2-hydroxyvaleric acid	0.247	0.0038
	Acylcarnitine C18:2	0.241	0.0061
	Uric acid	0.234	0.0063
**NT-proBNP**	2-aminobutyric acid	-0.628	0.0001
	LPC (20:4)	0.603	0.0001
	Indole-3-acetate	0.494	0.0001
	PC(18:1/20:3)	-0.478	0.0001
	Cysteine-glycine	-0.466	0.0001
	LPC (22:6)	-0.401	0.0001
	PC(18:2/20:5)	-0.352	0.0001
	LPC (18:0)	-0.306	0.0101
	Pyruvic acid	0.299	0.0001
	Acylcarnitine C10:0	0.27	0.0003
	CE (18:0)	0.193	0.0029
	DG(16:0/16:0)	-0.163	0.0142

Standardized regression coefficient indicates the impact of one individual predictor over the specific test if all other predictors remain constant. Expected low values of exercise duration, OUES, and Peak VO_2_ and high values of VEVCO_2_, NT-proBNP, Galectin-3, and CRP predict poor test performance. Negative coefficient values are indicative of inverse correlation. CPET = cardiopulmonary exercise testing; Peak VO_2_ = peak oxygen consumption; OUES = oxygen uptake efficiency slope; VE/VCO_2_ = minute ventilation-carbon dioxide production; NT-proBNP = N-terminal pro-B-type natriuretic peptide; CRP = C-reactive protein; PC = phosphatidylcholine; DG = diacylglycerol; PI = phosphatidylinositol; Cer = ceramide; CE = cholesteryl ester; FA = fatty acid; LPC = lysophosphatidylcholine.

From the regression models it was possible to estimate the mean response of CPET and HFBio (Table 1) use the metabolites predictors to estimate the HF test performance. Therefore, the metabolic modulation can be used to estimate the HF tests mean response as a threshold cut-off. HF test response above or below this estimated cut-off are interpreted as HF patients’ good or poor performance and of outcome prognosis. In Table 1 it is shown that the HF tests mean response are comparable with HF tests’ threshold references published by other authors. To be able to compare models we used the coefficient of variance, where the model with the smaller CV has predicted values that are closer to the actual values. Based in the metabolites prediction, we found that VE/VCO_2_ and Peak VO_2_ (CV = 0.07 and 0.08, respectively) were the best predictive model for HF test performance and HF prognosis. Galectin-3 (CV = 0.13) also showed a reasonable predictive model, while CRP and NT-ProBNP (CV = 0.31 and 0.29, respectively) were the least fit models. This results indicates that CPET can be reasonably explained by their metabolites predictors, while HFbio are weekly explained.

**Fig 3:**
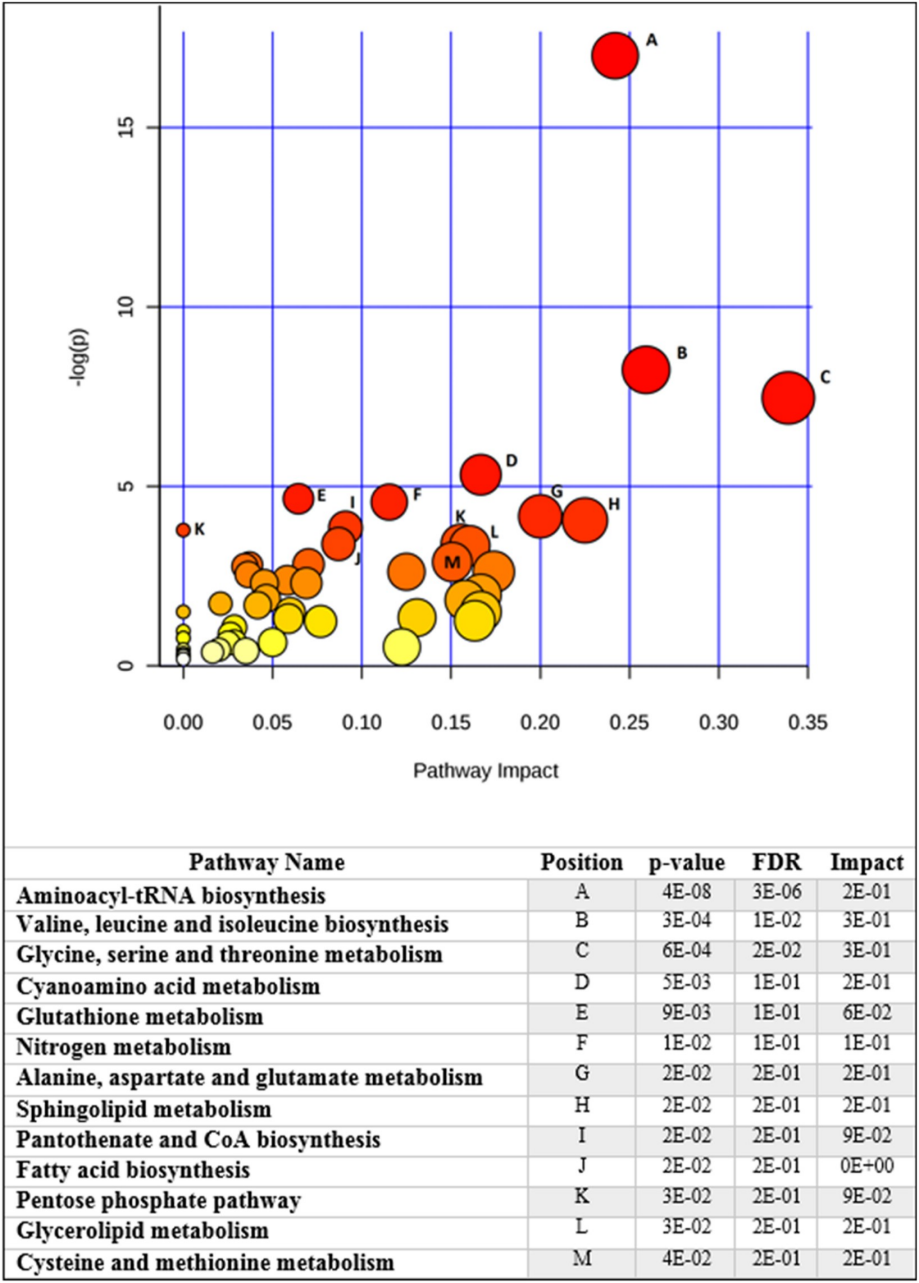
Metabolic pathway enrichment analysis shows the main pathways involved in heart failure test performance. The plot shows matched pathways according to the p-values from the pathway enrichment analysis and pathway impact values from pathway topology analysis. The pathways with the lowest p-values and highest match status (predictors present in the pathways) are listed in the table along with their FDR correction and impact score.

The diverse metabolites and lipids species compounding the CPET and HFBio prediction models are suggestive of the metabolic profile of the HF patient’s cohort. Therefore, they were used in the pathway enrichment analysis to indicate the more significant pathways impacting the prediction of HF patient’s performance. Fig 3 shows that 13 pathways are significantly involved in prediction of HF test performance. Aminoacyl-tRNA, amino acid biosynthesis, amino acid metabolism, nitrogen metabolism, pantothenate and CoA biosynthesis, along with sphingolipid and glycerolipid metabolism, fatty acid biosynthesis, as well as glutathione metabolism and pentose phosphate pathway were revealed as the more relevant pathways.

**Fig 4:**
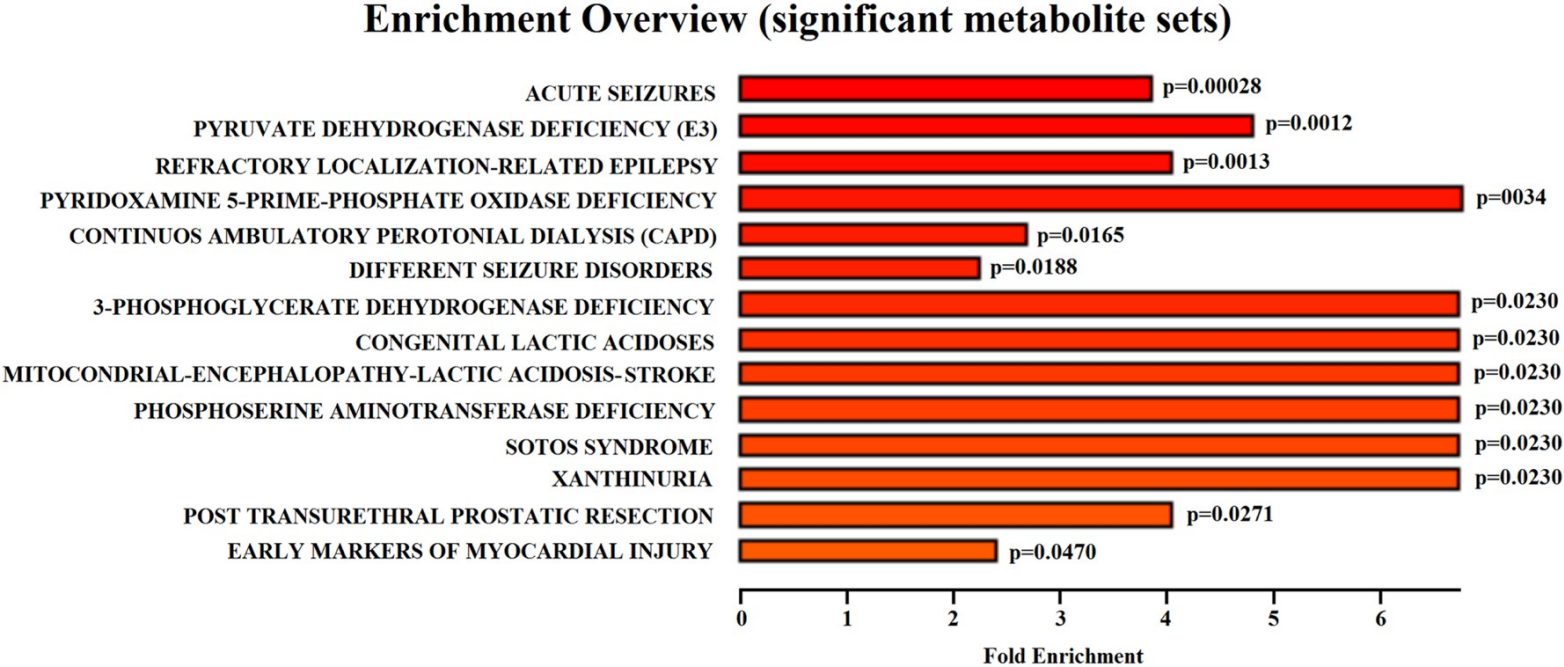
Diseases associated sets enrichment shows the more important diseases presenting similar metabolic profile based in heart failure test performance. The majority of the statistically significant enriched diseases are related to brain dysfunction. Lactic acidosis-related diseases were also found with high impact in the analysis.

To explore the similarity of other diseases metabolic dysfunction to HF metabolic modulation, a pathway enrichment analysis was utilized (Fig 4). This analysis revealed 14 disorders statistically significant and with high impact that demonstrated similar metabolic perturbation to that observed by us for HF. Most of these identified diseases were related to brain dysfunction, such as acute seizure disorders and epilepsy-like metabolic profiles indicating the compromise of brain function. Enzymes deficiencies were also detected associated to lactic acidosis. Peritoneal dialysis and early markers of myocardial injury were present with lower impact. These results indicate that the metabolic profile of HF patients is mostly similar to brain- like dysfunction, and renal and cardiac abnormalities.

**Fig 5:**
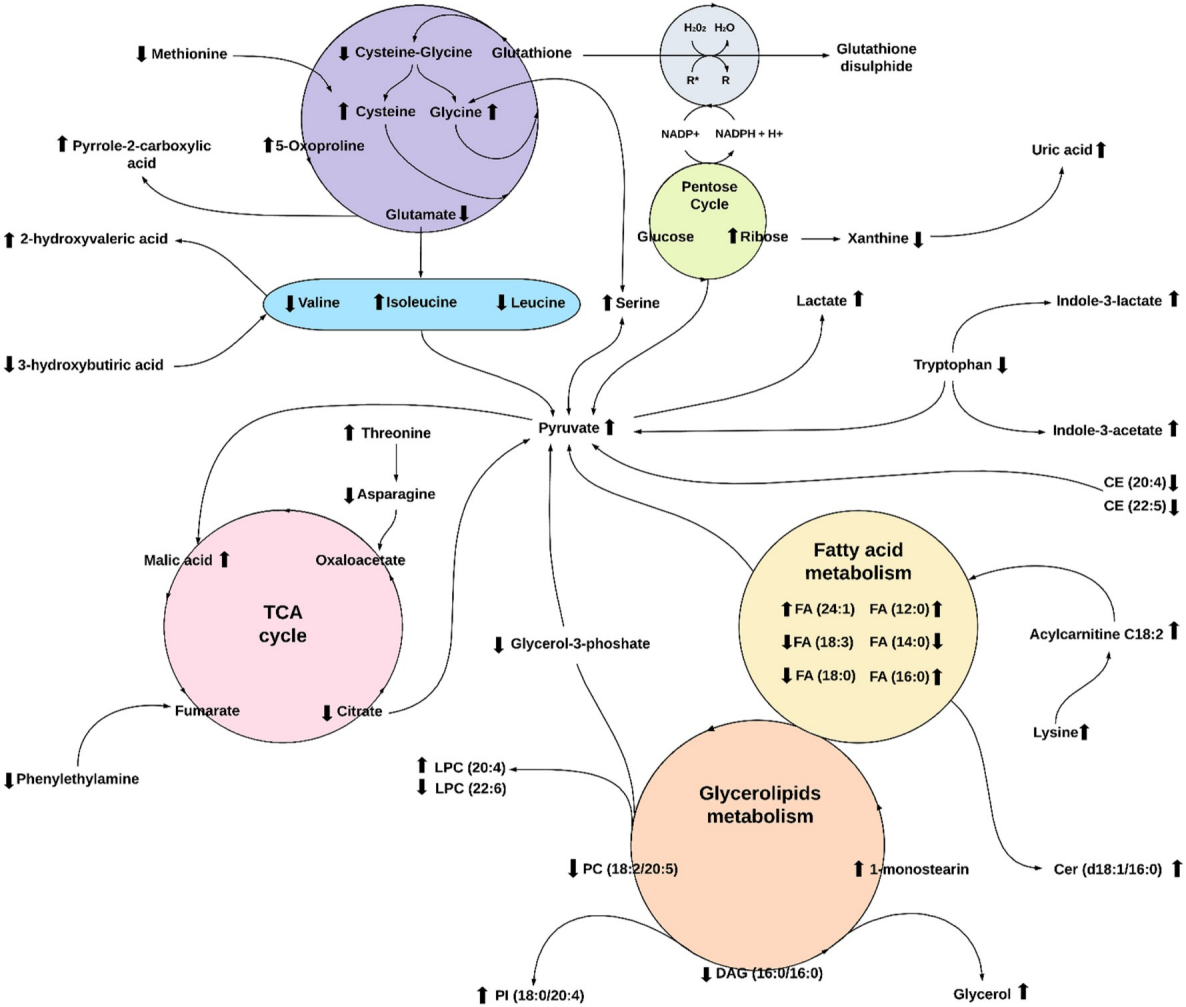
Intertwined metabolic network in heart failure. The metabolic changes affecting heart failure patients based in heart failure test performance includes glutathione anti-oxidative pathway, branched-chain amino acid (BCAA) biosynthesis, pentose cycle, tricarboxylic acid cycle (TCA), fatty acid (FA) metabolism, sphingolipids and glycerophospholipids metabolism, and tryptophan metabolism. Arrow represents predicted elevation or decrease variables in poor test performance. Only predictors with coefficients higher than 0.3 were used. Some metabolites not detected in the analysis were included in the figure to complement the metabolic pathways. Direction of pathways are proposed based in the metabolic modulation found in the study. TCA = tricarboxylic acid; PC = phosphatidylcholine; DAG = diacylglycerol; PI = phosphatidylinositol; Cer = ceramide; CE = cholesteryl ester; FA = fatty acid; LPC = lysophosphatidylcholine R* = reactive oxygen species.

Predictors of HF tests performance, supported by pathway enrichment analysis, were also used to propose a metabolic network suggestive of the metabolic modulations associated with HF test performance (Fig 5). In the proposed network, the positive or negative signs of the models’ coefficients were used as indication of metabolites elevation or decrease related to the expected poor test performance, respectively. The metabolic network was built linking relevant pathways revealed in the study, and the direction of pathways were suggested by metabolites elevation or decrease.

We chose to base our analyses focused in the metabolic modulations in poor test performance as an indication of HF poor prognosis. Our results indicates an overall profile of oxidative stress, lactic acidosis, and metabolic syndrome, coupled with mitochondria dysfunction. There are signs of glutathione depletion, represented by decreased cysteine-glycine dipeptide and glutamate, and elevation of methionine, cysteine, glycine, and 5-oxoproline. The proposed oxidative stress could induce ribose elevation from the pentose phosphate pathway (PPP) and decreased xanthine and consequent elevation of uric acid in poor performance. Glutamate catabolism is indicated by elevation of downstream products such as pyrrole-2-carboxilic acid and hydroxyproline dipeptide.

In our study, poor performance is associated with low levels of the branched-chain amino acids (BCAAs) leucine and valine, except elevation of isoleucine. Other amino acids modulations are elevated threonine and serine and decreased asparagine. Elevation of a compound similar to metabolites of BCAA catabolism, 2-hydroxyvaleric acid, was also detected. We also detected consumption of 3-hydroxybutyric acid, a ketone body linked to BCAAs metabolism, and decreased tryptophan levels. It appears that tryptophan catabolism is leading to elevation of indole-3-acetate and indole-3-lactate in an oxidative stressed environment.

Pyruvate was also elevated in poor performance. The decreased citrate in TCA cycle suggest that anaplerotic reactions are present. Therefore, the destiny of accumulated pyruvate could be responsible for malic acid elevation, but also responsible for elevation of lactate indicating lactic acidosis as a metabolic profile of poor HF test performance. Also in a link to fumarate in the TCA cycle, there was a decrease of phenylethanolamine.

Signs of fatty acids β-oxidation was inferred from the decreases in myristic acid (14:0), stearic acid (18:0), and linolenic acid (18:3). However, we found the levels of lauric acid (12:0), palmitic acid (16:0), and nervonic acid (24:1) were elevated in poor performance indicating special functions for these fatty acids. A sign that transport of fatty acids through the mitochondria membrane for β-oxidation is affected is the detected elevation of acylcarnitine C18:2 and lysine, a carnitine precursor, in the plasma. The analysis also suggests decreased levels of dietary polyunsaturated fatty acids corresponding to decreased levels of cholesteryl ester CE (20:4) and CE (22:5).

In the glycerolipids metabolism, elevation of lysophosphatidylcholine (LPC) carrying arachidonic acid (AA) and decrease of LPC carrying docosahexaenoic acid (DHA) suggests an imbalance toward accumulation of phospholipids containing pro-inflammatory fatty acids. This scenario is also supported by the detected decrease of phosphatidylcholine carrying eicosapentaenoic acid (EPA), PC (18:2/20:5), and elevation of phosphatidylinositol (PI) species containing omega-6 fatty acids represented by PI (16:0/18:2) and PI (18:0/20:4). We also observed decrease of DAG (16:0/16:0) that could be associated with the elevation of monoacylglycerol 1-monostearin and glycerol.

### Discussion

The small sample size of the cohort carried the risk of overfitting and misinterpretation of the regression estimates. In order to overcome this challenge, the small sample size was extensively dealt with in the statistical validation component of the study. The standardization of the regression coefficients is a mandatory step that help to decrease the impact of the small sample size. We also used the adjusted R^2^ as a mean to deal with the small sample size. The adjusted R^2^ compares the sample size to the number of terms in the regression to produce unbiased estimators of the population.

Another accepted approach for dealing with small sample size problem is the actual physiological and biochemical reasonability of the modulation of the identified metabolic predictors. To help in interpreting this metabolic modulation suggested by our HF tests performance predictive models we used metabolic pathways enrichment methods. We found that the results shows several significant metabolic pathways (Fig 3), also similar to known metabolic pathways involved in diseases that could be associated with the outcome of leaving with heart failure pathology (Fig 4). This analysis gave support to a comprehensive metabolic network that we suggest in Fig 5.

Our analysis observed the presence of metabolic modulation dysfunction, characterized by indicators of oxidative stress, lactic acidosis, and amino acid and lipid metabolic disorders, within HF patients with poor cardiorespiratory fitness and unfavorable prognosis according to traditional HF biomarkers. It is known that patients with diabetes, hypertension, and hyperlipidemia presented a higher risk of oxidative stress and metabolic disorders due to decreased antioxidant defenses[24]. There is also a relationship between plasma total cysteine (tCys) and the risk of cardiovascular diseases and vascular toxicity of cysteine[25,26], as the fast auto-oxidation of cysteine can generate reactive oxygen species (ROS)[27]. High levels of cysteine and glycine are also associated with an elevated risk of developing metabolic syndrome[28]. A decreased methionine level is critical for anti-oxidative processes since methionine is a direct target of ROS, acting as a scavenger of free radicals[29,30]. Moreover, accumulation of 5-oxoproline in the blood could be responsible for metabolic acidosis associated with oxidative stress[31,32]. The reduction of glutamate can affect the brain’s activity, since glutamate acts as an excitatory neurotransmitter binding to the N-methyl-D-aspartate receptors and activate chloride ion channels[33].

As stated previously, it has been demonstrated that a reduction in the efficacy of amino acid metabolic activity with regards to poor test performance. Congestive HF (CHF) patients have reduced arterial amino acids that are related to HF severity[34]. Defective BCAAs catabolism and the elevation of branched-chain α-keto acids have also been associated with HF[35]. 2-hydroxyisovaleric acid has been reported in urine of patients with keto and lactic acidosis[36], as well as 3-hydroxybutyric acid has being found in patients with CHF[37]. Also, tryptophan degradation and elevation of indole-3-acetate suggest pro-inflammatory and pro-oxidant effects[38].

Pro-inflammatory and pro-oxidant effects were not the only physiological attributes we found. Our study also revealed that phenylethanolamine metabolism also was affected in poor performance. This metabolite is usually recognized as part of the phenylethanolamine N-methyltransferase (PNMT), an enzyme that converts norepinephrine to epinephrine. In a mouse model with knocked out PNMT, epinephrine-deficient’ mice had an exaggerated blood pressure response to exercise and reduced cardiac filling, indicating that epinephrine is required for maintaining normal cardiovascular function during stress[39].

Along with PNMT, elevated levels of fatty acid oxidation are a common metabolic disturbance in HF and other cardiopathies[40]. Activation of compensatory mechanisms such as PPP play a critical role in regulating cellular oxidative stress and lipids synthesis, although it can paradoxically feed superoxide-generating enzymes[41,42]. Ribose catabolism was linked to uric acid elevation in poor performance, and uric acid was associated with markers of metabolic syndrome and of systematic inflammation[43,44].

We detected a reduction of citrate and an elevation of malic acid in the TCA cycle, supporting the observation that the carboxylation of pyruvate to malate is an important anaplerotic reaction in HF[45]. In a study of hypertrophied rat hearts, the literature found that the rate of palmitic acid entering into oxidative metabolism was reduced by 23% compared to normal heart metabolic activity; the reduced rate of palmitate oxidation was balanced by a compensatory increase in anaplerotic flux, and the increased anaplerosis in hypertrophic hearts was fueled in part by increased carboxylation of the glycolytic pyruvate[46].

The elevated pyruvate in poor performance could be explained by the activation of fatty acid oxidation to produce acetyl-CoA, causing pyruvate oxidation inhibition in ischemic organs and driving pyruvate conversion to lactate[47]. Notwithstanding the detected oxidation of fatty acids, we found the levels of lauric acid (12:0), palmitic acid (16:1), and nervonic acid (24:1) were elevated in poor performance. Lauric acid elevation has being associated with an increased risk of coronary disease and ischemic stroke[48]. Palmitic acid is one of the more abundant fatty acids in human plasma and was the predictor with the highest impact for CRP. Wu *et al* reported that palmitic acid significantly stimulated in vitro CRP, TNF-α, and iNOS expression at the mRNA and protein levels in vascular smooth muscle cells[49]. Nervonic acid, a long-chain monounsaturated omega-9 fatty acid that undergoes β-oxidation in peroxisomes, plays an important role as an intermediate in the biosynthesis of nerve cell myelin[50]. Higher levels of nervonic acid have been positively associated with greater congestive HF and an increased risk of cardiovascular mortality, suggesting nervonic acid may pose as a cardiotoxin in humans[51]. Fatty acids like nervonic acid again have shown a significant role in HF modulation.

By the detection of elevated plasma acylcarnitine, we can infer that modulated fatty acids involved in HF β-oxidation are transported through the mitochondrial membrane. In studying the association of metabolites with adverse HF outcomes, Ahmad *et al* found that plasma acylcarnitine C18:2 was significantly higher in patients with end-stage HF[52]. The metabolic derangement in poor performance is also supported by the quantifiable differences in sphingolipid, glycerolipid, and glycerophospholipid metabolisms. In a rat model, myocardial Cer (d18:1/16:0) increased significantly 24 hours after acute myocardial infarction[53]. Elevation of pro-inflammatory lipids such as the specific LPC carrying arachidonic acid (AA) suggests an imbalanced accumulation of phospholipids containing omega-6 pro-inflammatory fatty acids[54]. This imbalance is further apparent by the presence of PC (18:2/20:5) and in our data with higher levels of phosphatidylinositol species containing omega-6 fatty acids PI (16:0/18:2) and PI (18:0/20:4). Phosphatidylinositol (PI) is especially abundant in brain tissue, along with a high AA content[55]. Biosynthesis of PI is linked to the reversible diacylglycerol (DAG) metabolism. DAG regulates the activity of protein kinase C that controls many key cellular functions, including ROS production[56]. Degradation of DAGs could be explained by the degradation of glycerolipids and glycerophospholipids from exacerbated oxidative stress[57].

### Limitations

This study has some limitations. First, the small cohort’ sample size does not permit a statistical hypothesis test that the predictors in the model has a real causality with the dependent variables. However, since the study was focused in finding predictive models that could reveal the metabolic modulation underlying the HF tests, the sample size was adequate for the task, but we could not add important covariates to the models as sex, age, and left ventricular ejection fraction categorizations. Second, the cohort was part of a comparative study of the effects of different doses of Anakinra in HF patients, and we used only the baseline data before the random group assignment, so we could evaluate the metabolic modulation without treatment, but limited the study power to compare with healthy matched control group. Third, the population demographic is characteristic of the single center facility where African American patients with high levels of diabetes, hyperlipidemia and hypertension are prevalent. This limited the extrapolation of our findings to this particular HF subpopulation, and suggest that further studies should be performed including broader demographic population sampling.

### Conclusion

Through our examination of metabolic dysfunction and HF patient test performances coupled to multiple regression research and analyses, we explored the predictive relationships among variables with respect to physiological context. In our study, MRA successfully demonstrated patterns of relationships that are consistent with interpretations reported by other authors, revealing the metabolic modulation associated with HF tests performance. Further studies using MRA with consideration to preexisting literature on physiology therefore could yield promising results, such as those found in this manuscript that could help improve the management of diseases and disorders.

## Supporting information

S1 Fig Predicted models of CPET and HFbio shows high correlation with metabolic modulation. The regression models of HF tests are plotted by actual test values per predict values, and the main estimates are represented. The modulation of HF tests’ predictors are plotted as well and the main response is highlighted in red, followed by respective 95% confidence intervals. All models rendered a predictive R^2^ higher than 0.7 in cross-validation. Overall, 73 predictors were found correlated with HF tests performance.
(TIFF)
